# Efficacy and Safety of Larotrectinib in Patients With Tropomyosin Receptor Kinase Fusion–Positive Lung Cancers

**DOI:** 10.1200/PO.21.00418

**Published:** 2022-01-27

**Authors:** Alexander Drilon, Daniel S. W. Tan, Ulrik N. Lassen, Serge Leyvraz, Yongmei Liu, Jyoti D. Patel, Lee Rosen, Benjamin Solomon, Ricarda Norenberg, Laura Dima, Nicoletta Brega, Lin Shen, Victor Moreno, Shivaani Kummar, Jessica J. Lin

**Affiliations:** ^1^ Memorial Sloan Kettering Cancer Center, New York, NY; ^2^ Weill Cornell Medical College, New York, NY; ^3^ Division of Medical Oncology, National Cancer Centre Singapore, Duke-NUS Medical School, Singapore; ^4^ Department of Oncology, Rigshospitalet, Copenhagen, Denmark; ^5^ Charité – Universitätsmedizin Berlin, Berlin, Germany; ^6^ Cancer Center and State Key Laboratory of Biotherapy, West China Hospital, Sichuan University, Chengdu, China; ^7^ Northwestern University, Chicago, IL; ^8^ UCLA Division of Hematology-Oncology, Los Angeles, CA; ^9^ Avera Cancer Institute, Sioux Falls, SD; ^10^ Chrestos Concept GmbH & Co KG, Essen, Germany; ^11^ Bayer HealthCare Pharmaceuticals, Inc, Basel, Switzerland; ^12^ Bayer HealthCare Pharmaceuticals, Whippany, NJ; ^13^ Department of Gastrointestinal Oncology, Key Laboratory of Carcinogenesis and Translational Research (Ministry of Education/Beijing), Peking University Cancer Hospital & Institute, Beijing, China; ^14^ START MADRID-FJD, Hospital Fundación Jiménez Díaz, Madrid, Spain; ^15^ Stanford Cancer Center, Stanford University, Palo Alto, CA; ^16^ Department of Medicine, Massachusetts General Hospital, Boston, MA; ^17^ Harvard Medical School, Boston, MA S.K.'s current affiliation is Oregon Health & Science University, Portland, OR.

## Abstract

Larotrectinib is a highly selective and CNS-active tropomyosin receptor kinase (TRK) inhibitor that has demonstrated efficacy across TRK fusion–positive cancers, regardless of the tumor type. The aim of this study was to assess the efficacy and safety of larotrectinib in patients with TRK fusion–positive lung cancers.

**MATERIALS AND METHODS:**

Data from two global, multicenter, registrational clinical trials of patients treated with larotrectinib were analyzed: a phase II adult and young adult basket trial (NCT02576431) and a phase I adult trial (NCT02122913). The primary end point was objective response rate (ORR).

**RESULTS:**

By July 20, 2020, 20 patients with TRK fusion–positive lung cancer had been treated. The ORR by investigator assessment among 15 evaluable patients was 73% (95% CI, 45 to 92); one (7%) patient had a complete response, 10 (67%) had a partial response, three (20%) had stable disease, and one (7%) had progressive disease as best response. The median duration of response, progression-free survival, and overall survival were 33.9 months (95% CI, 5.6 to 33.9), 35.4 months (95% CI, 5.3 to 35.4), and 40.7 months (95% CI, 17.2 to not estimable), respectively. Among patients with baseline CNS metastases, the ORR was 63% (95% CI, 25 to 91). Adverse events were mainly grade 1 or 2.

**CONCLUSION:**

Larotrectinib is highly active with rapid and durable responses, extended survival benefit, and a favorable long-term safety profile in patients with advanced lung cancer harboring *NTRK* gene fusions, including those with CNS metastases. These findings support routine testing for *NTRK* fusions in patients with lung cancer.

## INTRODUCTION

The neurotrophic tyrosine receptor kinase genes (*NTRK*) *NTRK1*, *NTRK2*, and *NTRK3* encode the tropomyosin receptor kinase (TRK) proteins TRKA, TRKB, and TRKC, respectively. TRK proteins play an important role in neurodevelopment and postdevelopmental physiologic processes such as balance maintenance, appetite control, and pain perception.^1,2^ Fusions involving *NTRK1*, *NTRK2*, or *NTRK3* are oncogenic drivers that are found in a variety of adult and pediatric tumor types, including < 1% of non–small-cell lung cancers (NSCLCs).^3,4^ Structurally, these fusions are characterized by a 3′ end containing one of the three *NTRK* genes, including the full kinase domain, and an upstream partner gene in the 5′ position.^5^ In-frame activating fusions result in constitutive activation of the TRK kinase and uninterrupted downstream signaling that drives tumor development.^2^ In NSCLC, similar to ALK, ROS1, and RET fusions, TRK fusions are associated with adenocarcinomas from younger patients with a minimal or no history of cigarette smoking, although these fusions have been observed across a range of tumor histologies, ages, and smoking histories.^3^

**CONTEXT**

**Key Objective**
To our knowledge, we report the first analysis of the efficacy and safety of larotrectinib exclusively in patients with tropomyosin receptor kinase (TRK) fusion–positive lung cancer from a registrational data set.
**Knowledge Generated**
Larotrectinib was highly active in patients with TRK fusion–positive lung cancer, producing rapid, marked, and durable responses, including in patients with brain metastases. Larotrectinib had a favorable safety profile and was well tolerated.
**Relevance**
These findings validate TRK fusions as key therapeutic targets and underscore the need to include *NTRK* fusion testing as part of comprehensive molecular profiling in patients with lung cancer.

Larotrectinib is a first-in-class, highly selective, and CNS-active TRK inhibitor.^6-8^ Larotrectinib received tumor-agnostic approval for the treatment of adult and pediatric patients with TRK fusion–positive cancers in 2018^9^ on the basis of the robust and durable antitumor efficacy observed in a pooled analysis of three phase I and II trials.^6^ This efficacy was sustained after further follow-up with an expanded patient population.^7^ Larotrectinib showed a favorable safety profile; only 8% and 2% of patients required a dose reduction or permanent treatment discontinuation, respectively, because of an adverse event (AE).^7^

To date, however, the prospective efficacy and safety of larotrectinib solely in patients with TRK fusion–positive lung cancers have not been published. The aim of this analysis was to provide data on these outcomes in patients treated on this registrational program.

## MATERIALS AND METHODS

### Study Design

Patients were eligible for inclusion in this analysis if they had advanced lung cancer harboring an *NTRK* fusion and participated in one of two global, multicenter trials of larotrectinib: a phase II basket trial in adult and pediatric patients age ≥ 12 years (NAVIGATE, NCT02576431) and a phase I trial in adults age ≥ 18 years (NCT02122913). Complete methodologies for these studies have been described in a previous publication.^6^

Briefly, patients were eligible for inclusion in the NAVIGATE trial if they had a locally advanced or metastatic solid tumor, had an Eastern Cooperative Oncology Group (ECOG) performance status of 0-3, had adequate major organ function, and had received prior standard therapy appropriate for their tumor type and stage of disease or in the opinion of the investigator would be unlikely to tolerate or derive clinically meaningful benefit from appropriate standard-of-care therapy. Patients must have received a platinum-based doublet with or without maintenance therapy (continuation or switch maintenance), unless they declined chemotherapy.

Patients were eligible for inclusion in the phase I study if they had a locally advanced or metastatic solid tumor that had progressed or was nonresponsive to available therapies, were considered unfit for standard chemotherapy, or had a tumor for which no standard or available curative therapy exists, and had an ECOG performance status of 0-2. Patients with asymptomatic CNS metastases were eligible. Patients also had to have at least one measurable lesion as defined by RECIST version 1.1.^6^

In patients included from both studies, treatment consisted of single-agent larotrectinib administered at 100 mg twice daily in continuous 28-day cycles. Treatment beyond progression was permitted if the patient continued to benefit. TRK fusion status was determined by molecular testing in Clinical Laboratory Improvement Amendments–certified or similarly accredited laboratories. All study protocols were approved by the institutional review board or independent ethics committees and complied with the International Ethical Guidelines for Biomedical Research Involving Human Subjects, Good Clinical Practice guidelines, the Declaration of Helsinki, and local laws. All patients, or guardians for patients younger than age 18 years, provided written informed consent. The data cutoff for the current analysis was July 20, 2020.

### Study End Points

The primary end point for the combined analysis was objective response rate (ORR) on the basis of RECIST version 1.1. Secondary end points included the duration of response (DoR), progression-free survival (PFS), and overall survival (OS), on the basis of investigator assessment. The occurrence of AEs, including treatment discontinuation and dose modifications, was also assessed per National Cancer Institute Common Terminology Criteria for AEs, version 4.03.

### Study Assessments

Tumor assessment was conducted using computed tomography, magnetic resonance imaging, and clinical measurement. Tumors were assessed at baseline, every 8 weeks for 12 months, and then every 12 weeks thereafter until disease progression. Additional imaging was allowed at the investigator's discretion.

### Statistical Analysis

DoR, PFS, and OS were estimated using Kaplan-Meier analysis. 95% CIs were calculated using the Clopper-Pearson method. These events were measured as previously described.^7^

## RESULTS

### Patient Population

A total of 20 patients with lung cancers harboring an *NTRK* fusion were included in this analysis. Baseline patient characteristics are shown in Table 1. The median age was 48.5 years (range, 25.0-76.0 years), and 18 (90%) patients had an ECOG performance status of 0 or 1. Tumor histology was adenocarcinoma in 19 (95%) patients and neuroendocrine carcinoma in one (5%) patient. Half of the patients had baseline CNS metastases, two of whom were previously treated with brain radiotherapy (Data Supplement).

**TABLE 1.**
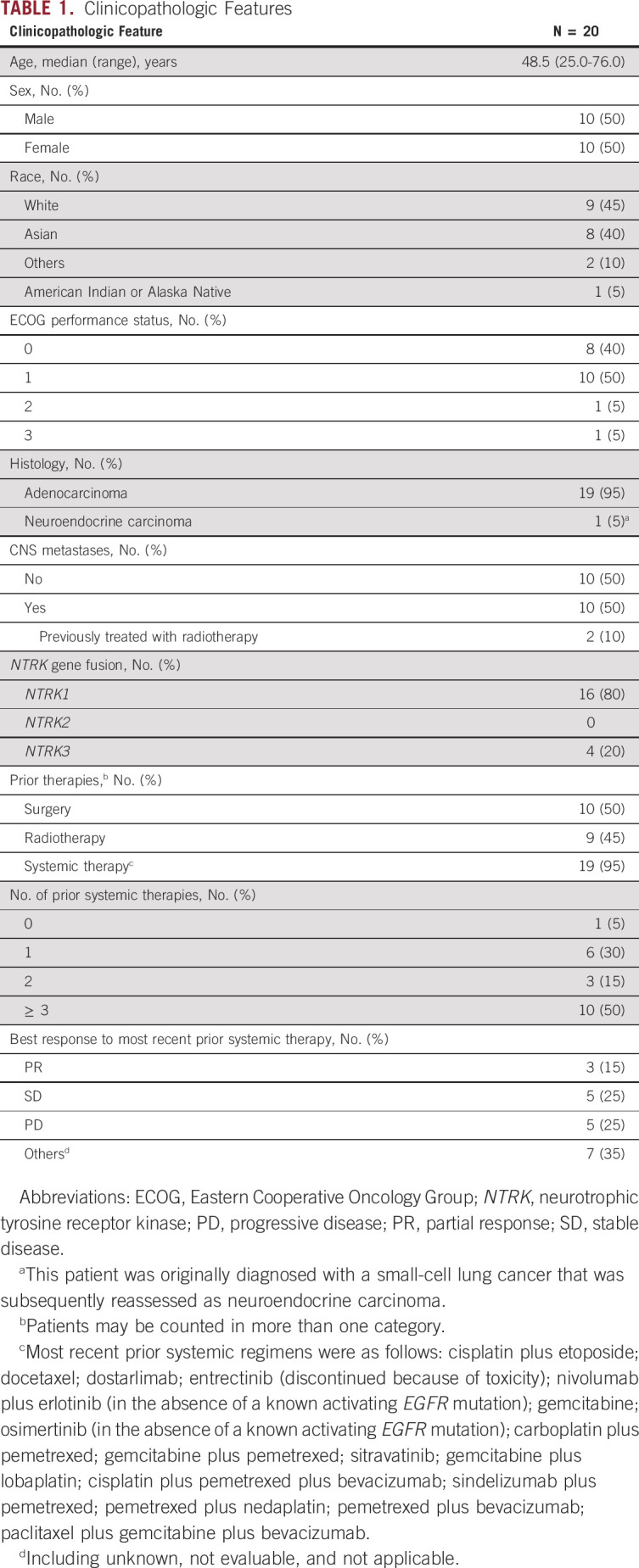
Clinicopathologic Features

*NTRK* fusions were identified by RNA-based sequencing in seven (35%) patients (four by anchored multiplex polymerase chain reaction, two by targeted next-generation sequencing [NGS], and one by whole transcriptome sequencing) and by targeted DNA-based NGS in 13 (65%) patients. Sixteen (80%) patients had fusions involving *NTRK1* (fusion partners: *TPM3* [n = 6], *EPS15* [n = 2], *IRF2BP2* [n = 2], *NOS1AP* [n = 1], *SQSTM1* [n = 1], *TPR* [n = 1], *CD74* [n = 1], *CLIP1* [n = 1], and *PRDX1* [n = 1]), and four (20%) patients had fusions involving *NTRK3* (fusion partners: *SQSTM1* [n = 2] and *ETV6* [n = 2]; Data Supplement).

Patients were heavily pretreated. The median number of prior lines of systemic therapy was three (range, 0-6), and 10 (50%) patients had received three or more prior systemic treatments (Data Supplement). Six (30%) patients had received prior immune checkpoint inhibitors.

### Efficacy

**FIG 1.**
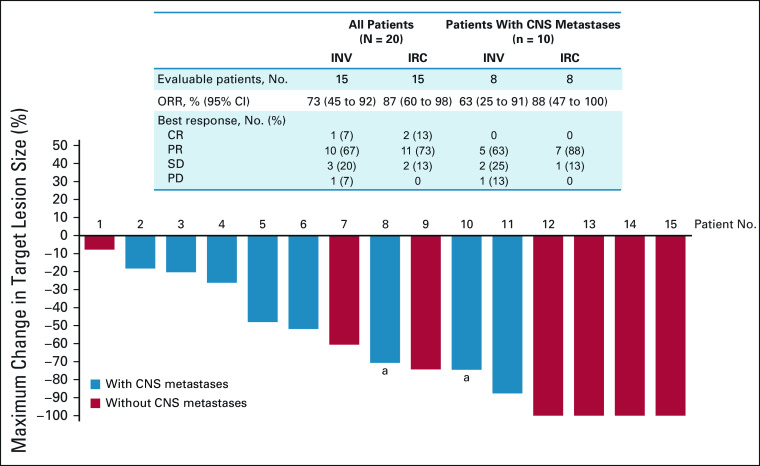
Response to larotrectinib. A waterfall plot of the maximum change in the target lesion size (investigator assessment) with larotrectinib treatment is shown for 15 evaluable patients whose lung cancers harbored a TRK fusion. ^a^Two patients had CNS metastases included as target lesions with a 100% and 59% reduction observed by cycle 4, respectively. Each patient number refers to the same patient across all tables and figures. CR, complete response; INV, investigator; IRC, independent review committee; ORR, objective response rate; PD, progressive disease; PR, partial response; SD, stable disease; TRK, tropomyosin receptor kinase.

**FIG 2.**
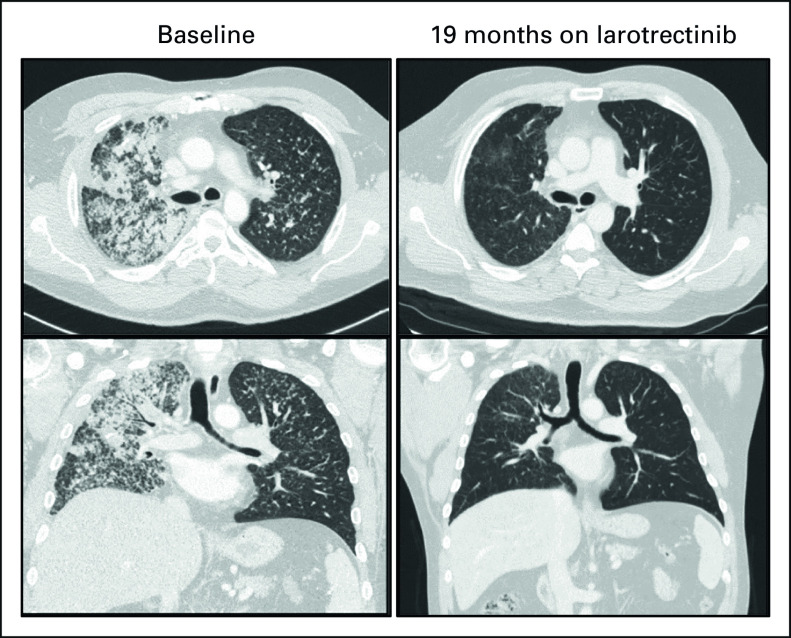
Response to larotrectinib. A 46-year-old man diagnosed with stage IV (T4N0M1) non–small-cell lung cancer previously progressed on first-line platinum-based chemotherapy (after 9 months) and on the PD-1 inhibitor dostarlimab (after 2 months). A *TPM3-NTRK1* fusion was identified on molecular profiling, and larotrectinib was initiated on trial. A brisk partial response was achieved at 1.8 months, which was subsequently confirmed; the patient discontinued treatment because of progressive disease after a durable 39 months of disease control. *NTRK*, neurotrophic tyrosine receptor kinase; PD-1, programmed cell death-1.

The investigator-assessed ORR among 15 evaluable patients was 73% (95% CI, 45 to 92); one (7%) patient had a complete response, 10 (67%) patients had a partial response, three (20%) patients had stable disease, and one (7%) had progressive disease (extracranial nontarget lesion) as the best response (Fig 1). The ORR by independent review committee assessment was consistent with the investigator-assessed results. The activity of larotrectinib in an exemplary responder is shown in Figure 2. Responses were achieved regardless of ECOG performance status, and a reduction in target tumor size was observed in all patients with measurable disease. Five of the 20 patients overall were not evaluable for response because they had not yet had a postbaseline tumor assessment.

**FIG 3.**
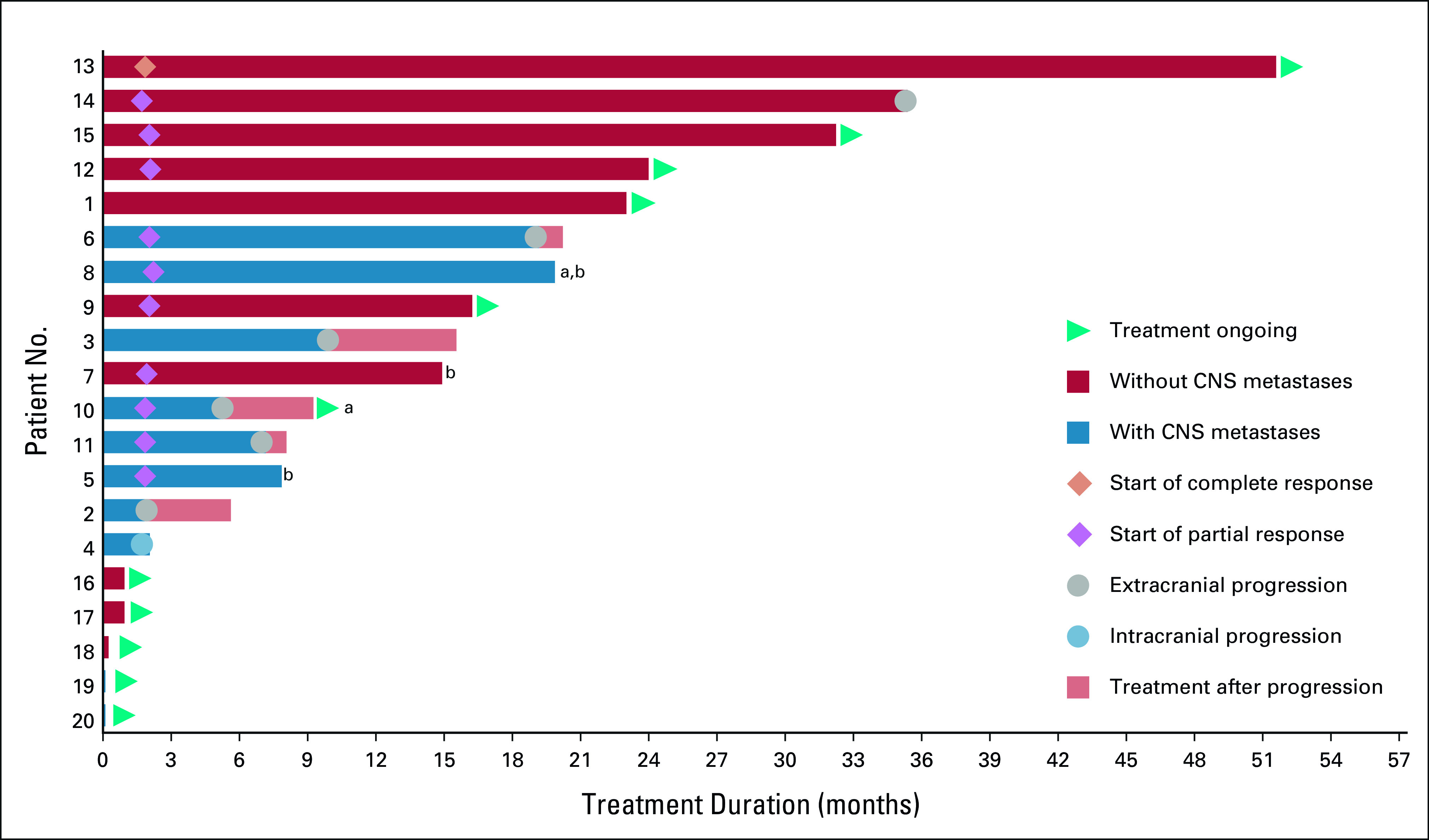
Larotrectinib treatment duration. A swimmer plot of the duration of larotrectinib treatment is shown for all 20 patients whose lung cancers harbored a TRK fusion. ^a^Two patients had CNS metastases included as target lesions with a 100% and 59% reduction observed by cycle 4, respectively. ^b^Patient discontinued for reasons other than progression. Each patient number refers to the same patient across all tables and figures. TRK, tropomyosin receptor kinase.

The duration of treatment ranged from 0.03+ to 51.5+ months. By the data cutoff, treatment was ongoing in 11 (55%) patients (Data Supplement). Among patients with an objective response (n = 11), the median time to response was 1.8 months (range, 1.6-1.9 months; Fig 3), corresponding to the first follow-up imaging examination on trial.

**FIG 4.**
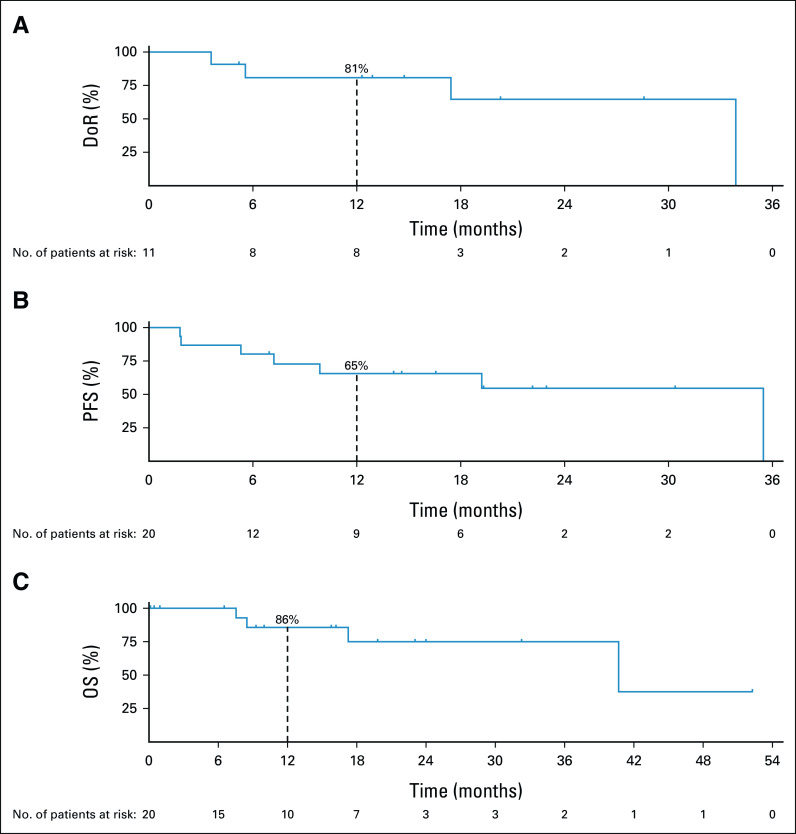
DoR and survival. Kaplan-Meier curves of (A) DoR, (B) PFS, and (C) OS are shown for patients with TRK fusion–positive lung cancers treated with larotrectinib. At the data cutoff, the median DoR, PFS, and OS were 33.9 months (95% CI, 5.6 to 33.9), 35.4 months (95% CI, 5.3 to 35.4), and 40.7 months (95% CI, 17.2 to NE), respectively. DoR, PFS, and OS rates at 12 months are indicated with vertical dashed lines on each curve. DoR, duration of response; NE, not estimable; OS, overall survival; PFS, progression-free survival; TRK, tropomyosin receptor kinase.

The median DoR was 33.9 months (95% CI, 5.6 to 33.9) at a median follow-up of 17.4 months; the DoR rates at 12 and 24 months were 81% and 65%, respectively. At a median follow-up of 16.6 months, the median PFS was 35.4 months (95% CI, 5.3 to 35.4). The 12- and 24-month PFS rates were 65% and 55%, respectively. Among 19 patients who had prior systemic therapy, 11 (58%) had > 2-fold longer PFS on larotrectinib compared with the time to progression or treatment failure on their most recent prior therapy (Data Supplement). Of the eight patients with < 2-fold longer PFS, seven were still on treatment and censored at the data cutoff. The median OS was 40.7 months (95% CI, 17.2 to not estimable) at a median follow-up of 16.2 months; the 12- and 24-month OS rates were 86% and 75%, respectively (Fig 4).

### CNS Efficacy

All eight evaluable patients with CNS metastases at baseline had reductions in overall (systemic) target lesions, ranging from 18% to 88%. The ORR by investigator assessment was 63% (95% CI, 25 to 91); five (63%) patients had a partial response, two (25%) had stable disease, and one (13%) had progressive disease (Fig 1). Although intracranial response was not a study end point, CNS metastases were included as target lesions for two patients, with measured reductions in the CNS metastases of 59% and 100% by cycle 4, respectively. Neither of these patients previously received brain radiotherapy.

The duration of treatment among all 10 patients with CNS metastases ranged from 0.03+ to 20.2 months, with three patients still on treatment at the data cutoff. Five patients continued to receive treatment after extracranial-only progression with maintained disease control in the CNS (Fig 3).

### Safety

Treatment-related AEs were reported by 16 (80%) patients. AEs that occurred in ≥ 15% of patients are shown in Table 2; AEs were mostly grade 1 or 2, and there were no unexpected or new safety signals. Eight (40%) patients experienced a grade 3 treatment-emergent AE. Two (10%) patients experienced grade 3 AEs considered related to larotrectinib (hypersensitivity, increased weight, and myalgia). There was one grade 5 AE (cardiac arrest) that was not considered related to larotrectinib.

**TABLE 2.**
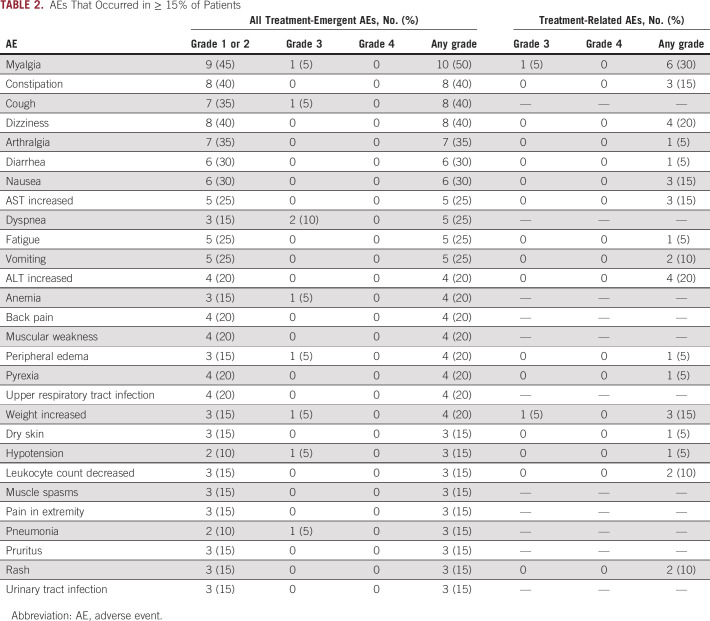
AEs That Occurred in ≥ 15% of Patients

Two (14%) patients required dose reductions because of AEs: grade 2 ALT and grade 2 AST increase in one patient (see the case report below), and grade 2 neutrophil count decrease in the other patient (all considered related to larotrectinib). Three (15%) patients had dose interruptions because of treatment-related AEs: grade 2 neutrophil count decrease in one patient, grade 2 AST and ALT increase in one patient, and grade 3 hypersensitivity in one patient. No patients experienced an AE that resulted in permanent discontinuation of larotrectinib.

## DISCUSSION

In this global, multicenter, registrational data set, larotrectinib was found to be highly active in patients with TRK fusion–positive lung cancers. The drug resulted in rapid, marked, and durable responses. The ORR by investigator assessment was 73%, and the median DoR, PFS, and OS were 33.9, 35.4, and 40.7 months, respectively. Furthermore, activity in the CNS was achieved. These results exceed the same outcome measures reported historically for chemotherapy^10^ and immunotherapy in NSCLCs. For example, first-line chemoimmunotherapy regimens have demonstrated a median PFS of 5.1-10.3 months and a median OS of 17.1-30.0 months.^11-17^

These data support the recommendation that a TRK inhibitor is the preferred up-front systemic therapy for advanced lung cancers that harbor a TRK fusion regardless of programmed cell death-ligand 1 expression levels, in line with current clinical practice guidelines. International expert consensus recommendations from the Japan Society of Clinical Oncology, European Society for Medical Oncology, ASCO, and the Taiwan Oncology Society strongly recommend the use of a TRK inhibitor during the course of therapy for any TRK fusion–positive cancer. Should a TRK fusion be discovered while a patient is already on another systemic therapy, switching to a TRK inhibitor on progression is recommended and can be considered if the response to the current systemic therapy is suboptimal.^18^

Similar to lung cancers in general,^19^ oncogene-driven lung cancers have a propensity for CNS metastasis. A multicenter registry previously reported that 36% of patients with TRK fusion–positive lung cancer had brain metastases,^3^ and prospective clinical trial data sets including this one have reported an incidence of 50%-69%.^20^ Larotrectinib demonstrated rapid and durable responses in patients with baseline brain metastases, consistent with previous findings from all larotrectinib-treated TRK fusion–positive cancers with baseline brain metastases where the ORR was 75%.^7^ In the latter data set, larotrectinib demonstrated intracranial activity in three patients with evaluable intracranial disease: complete intracranial disease resolution in a patient with NSCLC and intracranial tumor reductions of 14% and 46% in two patients with thyroid cancer.^7^ These findings support the use of larotrectinib in TRK fusion–positive cancers with brain metastases.

Another approved therapy for patients with TRK fusion–positive cancers is the multikinase inhibitor entrectinib, which showed an ORR of 69% in 13 patients with lung cancer and a median PFS and OS of 14.9 months at a median follow-up of 14.2 months.^20^

Larotrectinib was well tolerated in this series, and no new or unexpected safety signals were observed compared with the larger data set of all larotrectinib-treated TRK fusion–positive cancers,^7^ making the drug amenable to long-term use. As with any potent TRK inhibitor, occasional unique on-target AEs can occur; these include dizziness, weight gain, paresthesias, and TRK inhibitor withdrawal pain that providers should monitor in the clinic.^21^ Also consistent with the larger larotrectinib data set, local therapy extended the duration of disease control in TRK fusion–positive lung cancers with oligoprogression or solitary site progression. This is in line with guidelines for other oncogene-driven NSCLCs, which recommend targeted therapy continuation after local therapy for disease progression.^22^

The identification of numerous actionable drivers in lung cancer supports the use of appropriately designed, comprehensive NGS panels for molecular profiling. Clinical practice guidelines recommend routine testing for *EGFR* mutations, *ALK* fusions, and *ROS1* alterations^23-25^ and the inclusion of *NTRK1–3*, *ERBB2, MET*, *BRAF*, *KRAS*, and *RET* in NGS panels.^25^ Our analysis demonstrates that patients with TRK fusion–positive lung cancers had better outcomes on larotrectinib compared with prior systemic therapy, supporting early testing. Orthogonal confirmatory assays including pan-TRK immunohistochemistry are available,^26^ particularly in practice environments without payer coverage for NGS.^27^ Furthermore, the fact that DNA-based NGS may not detect all fusions^28^ supports the use of complementary RNA-based NGS to maximize the likelihood of identifying *NTRK* (in addition to *ALK*, *ROS1*, and *RET*) fusions.

Acquired resistance to TRK inhibitors may occur through on-target or off-target mechanisms. On-target resistance involves the development of TRK kinase domain mutations, which sterically restrict the binding of first-generation TRK inhibitors. Next-generation TRK inhibitors with activity against TRK kinase domain resistance mutations are in clinical development.^8,29^ Off-target resistance mechanisms involve bypass signaling activation because of genomic alterations in other oncogenic drivers, and these alterations may often be targetable with existing agents.^30^

In summary, the highly selective TRK inhibitor larotrectinib is active and well tolerated in patients with TRK fusion–positive lung cancers, including those with CNS metastases. These findings validate TRK fusions as key therapeutic targets and underscore the need for TRK fusion–inclusive early molecular testing strategies in patients with lung cancer.
